# Dimethyl *trans*-3-(4-bromo­phen­yl)-2-methyl­isoxazolidine-4,5-dicarboxyl­ate

**DOI:** 10.1107/S1600536809032462

**Published:** 2009-08-22

**Authors:** Orhan Büyükgüngör, Serkan Yavuz, Mustafa Odabaşoğlu, Hamdi Özkan, Özgür Pamir, Yılmaz Yıldırır

**Affiliations:** a Department of Physics, Faculty of Arts & Science, Ondokuz Mayıs University, TR-55139 Kurupelit Samsun, Turkey; b Department of Chemistry, Faculty of Arts & Science, Gazi University, Ankara, Turkey; c Chemical Technology Program, Denizli Higher Vocational School, Pamukkale University, TR-20159 Kınıklı, Denizli, Turkey; d Department of Chemistry, Faculty of Arts & Science, Kırıkkale University, Kırıkkale, Turkey

## Abstract

In the title compound, C_14_H_16_BrNO_5_, the isoxazolidine ring adopts an envelope conformation, with the N atom at the flap. In the crystal, inter­molecular C—H⋯N and C—H⋯O hydrogen bonds generate *R*_3_^3^(18) ring motifs which are fused into a ribbon-like structure extending along the *b* axis.

## Related literature

For general background, see: Confalone & Huie (1988 ▶); Torssell (1988 ▶); Frederickson (1997 ▶); Gothelf & Jorgensen (1998 ▶); Chiacchio *et al.* (2003 ▶); Padwa *et al.* (1981 ▶, 1984 ▶); Ochiai *et al.* (1967 ▶); Baldwin & Aube (1987 ▶); Heaney *et al.* (2001 ▶). For hydrogen-bond motifs, see: Bernstein *et al.* (1995 ▶); Etter (1990 ▶). For ring conformations, see: Cremer & Pople (1975 ▶).
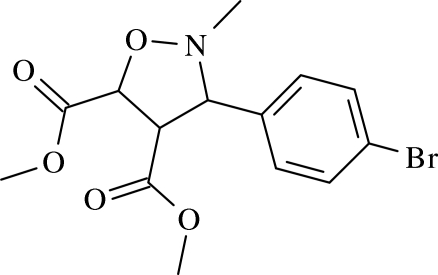


## Experimental

### 

#### Crystal data

C_14_H_16_BrNO_5_
*M* *_r_* = 358.19
Monoclinic, 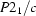

*a* = 10.9020 (4) Å
*b* = 8.1780 (3) Å
*c* = 17.8127 (8) Å
β = 101.622 (3)°
*V* = 1555.56 (11) Å^3^
*Z* = 4
Mo *K*α radiation
μ = 2.67 mm^−1^
*T* = 296 K
0.71 × 0.60 × 0.45 mm

#### Data collection

Stoe IPDS II diffractometer
Absorption correction: integration (*X-RED32*; Stoe & Cie, 2002 ▶) *T*_min_ = 0.327, *T*_max_ = 0.480
15678 measured reflections
3232 independent reflections
2696 reflections with *I* > 2σ(*I*)
*R*_int_ = 0.043

#### Refinement

*R*[*F* ^2^ > 2σ(*F* ^2^)] = 0.044
*wR*(*F* ^2^) = 0.101
*S* = 1.12
3232 reflections
193 parameters
H-atom parameters constrained
Δρ_max_ = 0.49 e Å^−3^
Δρ_min_ = −0.93 e Å^−3^

### 

Data collection: *X-AREA* (Stoe & Cie, 2002 ▶); cell refinement: *X-AREA*; data reduction: *X-RED32* (Stoe & Cie, 2002 ▶); program(s) used to solve structure: *SHELXS97* (Sheldrick, 2008 ▶); program(s) used to refine structure: *SHELXL97* (Sheldrick, 2008 ▶); molecular graphics: *ORTEP-3 for Windows* (Farrugia, 1997 ▶); software used to prepare material for publication: *WinGX* (Farrugia, 1999 ▶).

## Supplementary Material

Crystal structure: contains datablocks I, global. DOI: 10.1107/S1600536809032462/ci2876sup1.cif

Structure factors: contains datablocks I. DOI: 10.1107/S1600536809032462/ci2876Isup2.hkl

Additional supplementary materials:  crystallographic information; 3D view; checkCIF report

## Figures and Tables

**Table 1:** Hydrogen-bond geometry (Å, °)

*D*—H⋯*A*	*D*—H	H⋯*A*	*D*⋯*A*	*D*—H⋯*A*
C3—H3⋯N1^i^	0.93	2.56	3.492 (4)	179
C12—H12*C*⋯O1^ii^	0.96	2.52	3.434 (5)	158

Symmetry codes: (i) 
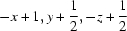
; (ii) 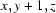.

**Fig. 1.**
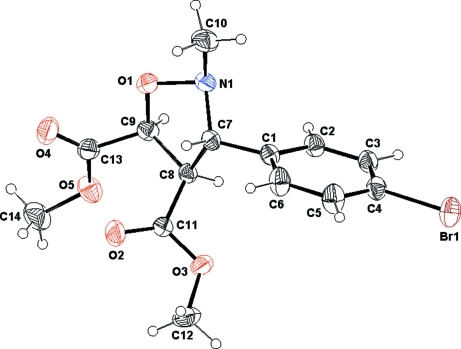
A view of (I) with the atomic numbering scheme. Displacement ellipsoids are drawn at the 30% probability level.

**Fig. 2.**
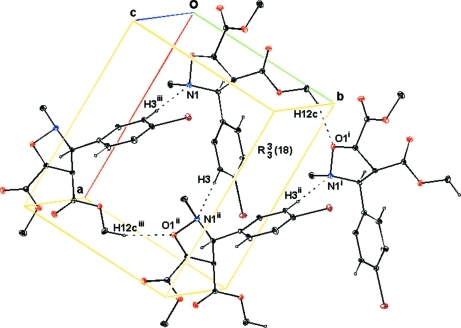
Part of the crystal structure of (I), showing the formation of hydrogen-bonded R33(18) motifs. H atoms not involved in hydrogen bonds have been omitted for clarity. Dashed lines indicate hydrogen bonds. [Symmetry codes: (i) x, 1 + y, z; (ii) 1 - x, 1/2 + y, 1/2 - z; (iii) 1 - x, y - 1/2, 1/2 - z].

**Fig. 3.**
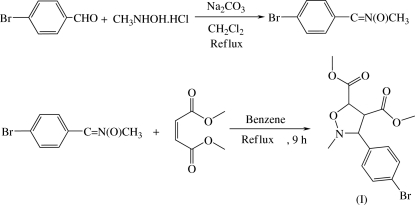
Preparation of the title compound.

## supplementary crystallographic information

### Comment

The 1,3-dipolar cycloaddition of nitrones and alkenes is a powerful synthetic device that allows up to three new stereogenic centers to be assembled in a stereospecific manner in a single step (Confalone & Huie, 1988; Torssell, 1988; Frederickson, 1997; Gothelf & Jorgensen, 1998). Among these N and O containing five-membered heterocycles, isoxazolidines and isoxazoline derivatives have emerged as important candidates and have been shown to display useful anticancer and antiviral properties (Chiacchio *et al.*, 2003).

The syntheses of isoxazolidine derivatives is an important subject in organic chemistry because they are found in the structure of most natural compounds and drugs. In recent years, isoxazolidine derivatives have been synthesized in high yield *via* intermolecular cycloaddition of *N*-methylnitrone with disubstituted olefins and are employed for biological evaluation.

These isoxazolidines are used in the syntheses of β-lactams (Padwa *et al.*, 1981) which are of value in the treatment of bacterial infections (Ochiai *et al.*, 1967), occur as natural products (Baldwin & Aube, 1987), serve as versatile synthetic intermediates (Padwa *et al.*, 1984), and are biologically interesting compounds. In view of the interest shown in these compounds, we report herein the crystal structure of the title compound, (I).

The overall view and atom-labelling of the molecule of (I) are displayed in Fig. 1. The isoxazolidine ring (O1/N1/C7-C9) adopts an envelope conformation, with atom N1 displaced by 0.326 (2) Å from the plane of the other ring atoms (Cremer & Pople, 1975).

The crystal packing is stabilized by intermolecular C—H···N and C—H···O hydrogen bonds (Table 1). As shown in Fig. 2, these hydrogen bonds form *R*_3_^3^(18) motifs which are fused to form ribbon-like structure extending along the *b* axis.

### Experimental

*N*-Methyl-*C*-(4-bromophenyl)nitrone was prepared from 4-bromo benzaldehyde, *N*-methyl-hydroxylamine hydrochloride and sodium carbonate in CH_2_Cl_2_ according to the literature method (Heaney *et al.*, 2001). For the preparation of the title compound, *N*-methyl-*C*-(4-bromophenyl) nitrone (453 mg, 3 mmol) and dimethylmaleate (475 mg, 3,3 mmol) were dissolved in benzene (50 ml). The reaction mixture was refluxed for 9 h, and monitored by TLC. After evaporation of the solvent, the reaction mixture was separated by column chromatography, using a mixture of hexane-ethyl acetate (1:2) as the eluent. The *trans*-isomer was recrystallized from methanol in 3 d (m.p. 354–355 K).

### Refinement

H atoms were positioned geometrically (C-H = 0.93–0.98 Å) and refined using a riding model with *U*_iso_(H) = 1.2*U*_eq_(C) and 1.5*U*_eq_(methyl C). A rotating–group model was used for the methyl groups.

### Crystal data

**Table:**

C_14_H_16_BrNO_5_	*F*(000) = 728
*M**_r_* = 358.19	*D*_x_ = 1.529 Mg m^−^^3^
Monoclinic, *P*2_1_/*c*	Mo *K*α radiation, λ = 0.71073 Å
Hall symbol: -P 2ybc	Cell parameters from 15678 reflections
*a* = 10.9020 (4) Å	θ = 1.9–28.0°
*b* = 8.1780 (3) Å	µ = 2.67 mm^−^^1^
*c* = 17.8127 (8) Å	*T* = 296 K
β = 101.622 (3)°	Block, colourless
*V* = 1555.56 (11) Å^3^	0.71 × 0.60 × 0.45 mm
*Z* = 4	

### Data collection

**Table:**

Stoe IPDS II diffractometer	3232 independent reflections
Radiation source: sealed X-ray tube, 12 x 0.4 mm long-fine focus	2696 reflections with *I* > 2σ(*I*)
plane graphite	*R*_int_ = 0.043
Detector resolution: 6.67 pixels mm^-1^	θ_max_ = 26.5°, θ_min_ = 1.9°
ω–scan rotation method	*h* = −13→13
Absorption correction: integration (*X-RED32*; Stoe & Cie, 2002)	*k* = −10→10
*T*_min_ = 0.327, *T*_max_ = 0.480	*l* = −22→22
15678 measured reflections	

### Refinement

**Table:**

Refinement on *F*^2^	Primary atom site location: structure-invariant direct methods
Least-squares matrix: full	Secondary atom site location: difference Fourier map
*R*[*F*^2^ > 2σ(*F*^2^)] = 0.044	Hydrogen site location: inferred from neighbouring sites
*wR*(*F*^2^) = 0.101	H-atom parameters constrained
*S* = 1.12	*w* = 1/[σ^2^(*F*_o_^2^) + (0.0399*P*)^2^ + 1.0321*P*] where *P* = (*F*_o_^2^ + 2*F*_c_^2^)/3
3232 reflections	(Δ/σ)_max_ = 0.001
193 parameters	Δρ_max_ = 0.49 e Å^−^^3^
0 restraints	Δρ_min_ = −0.93 e Å^−^^3^

### Special details

**Table:**

Geometry. All e.s.d.'s (except the e.s.d. in the dihedral angle between two l.s. planes) are estimated using the full covariance matrix. The cell e.s.d.'s are taken into account individually in the estimation of e.s.d.'s in distances, angles and torsion angles; correlations between e.s.d.'s in cell parameters are only used when they are defined by crystal symmetry. An approximate (isotropic) treatment of cell e.s.d.'s is used for estimating e.s.d.'s involving l.s. planes.
Refinement. Refinement of *F*^2^ against ALL reflections. The weighted *R*-factor *wR* and goodness of fit *S* are based on *F*^2^, conventional *R*-factors *R* are based on *F*, with *F* set to zero for negative *F*^2^. The threshold expression of *F*^2^ > σ(*F*^2^) is used only for calculating *R*-factors(gt) *etc*. and is not relevant to the choice of reflections for refinement. *R*-factors based on *F*^2^ are statistically about twice as large as those based on *F*, and *R*- factors based on ALL data will be even larger.

### Fractional atomic coordinates and isotropic or equivalent isotropic displacement parameters (Å^2^)

**Table:**

	*x*	*y*	*z*	*U*_iso_*/*U*_eq_	
C2	0.4201 (3)	0.5503 (4)	0.18030 (16)	0.0460 (7)	
H2	0.4149	0.4978	0.2259	0.055*	
C8	0.1314 (2)	0.4572 (3)	0.16330 (15)	0.0371 (5)	
H8	0.1754	0.5207	0.2072	0.044*	
C5	0.4354 (3)	0.7096 (5)	0.04589 (18)	0.0617 (9)	
H5	0.4399	0.7642	0.0007	0.074*	
C12	0.0010 (4)	0.8290 (4)	0.0637 (3)	0.0793 (12)	
H12C	0.0311	0.9380	0.0761	0.095*	
H12B	−0.0850	0.8216	0.0685	0.095*	
H12A	0.0074	0.8037	0.0120	0.095*	
C11	0.0347 (3)	0.5632 (3)	0.11402 (16)	0.0403 (6)	
O3	0.0753 (2)	0.7144 (3)	0.11549 (15)	0.0625 (6)	
C10	0.3202 (3)	0.1297 (5)	0.1037 (2)	0.0643 (9)	
H10C	0.3990	0.1723	0.0965	0.077*	
H10B	0.2642	0.1192	0.0549	0.077*	
H10A	0.3329	0.0243	0.1278	0.077*	
C4	0.5204 (3)	0.7411 (4)	0.11187 (18)	0.0473 (7)	
C6	0.3420 (3)	0.5947 (5)	0.04732 (18)	0.0575 (8)	
H6	0.2844	0.5715	0.0025	0.069*	
N1	0.2659 (2)	0.2410 (3)	0.15238 (14)	0.0448 (5)	
O1	0.14367 (19)	0.1690 (2)	0.15537 (13)	0.0506 (5)	
O2	−0.0615 (2)	0.5161 (3)	0.07465 (15)	0.0670 (7)	
C13	−0.0534 (3)	0.2653 (4)	0.17647 (19)	0.0497 (7)	
C14	−0.2378 (3)	0.3749 (6)	0.2055 (3)	0.0783 (12)	
H14C	−0.2637	0.4566	0.2378	0.094*	
H14B	−0.2667	0.2696	0.2183	0.094*	
H14A	−0.2728	0.3994	0.1529	0.094*	
O4	−0.1095 (2)	0.1619 (3)	0.1377 (2)	0.0846 (9)	
O5	−0.1029 (2)	0.3738 (3)	0.21707 (14)	0.0628 (6)	
Br1	0.64870 (3)	0.89981 (5)	0.11195 (2)	0.06568 (15)	
C3	0.5147 (3)	0.6630 (4)	0.17970 (17)	0.0462 (7)	
H3	0.5732	0.6857	0.2242	0.055*	
C7	0.2260 (2)	0.3988 (3)	0.11494 (15)	0.0385 (6)	
H7	0.1817	0.3788	0.0622	0.046*	
C9	0.0865 (3)	0.2939 (4)	0.19170 (16)	0.0432 (6)	
H9	0.1185	0.2863	0.2471	0.052*	
C1	0.3332 (2)	0.5142 (4)	0.11427 (15)	0.0400 (6)	

### Atomic displacement parameters (Å^2^)

**Table:**

	*U*^11^	*U*^22^	*U*^33^	*U*^12^	*U*^13^	*U*^23^
C2	0.0426 (14)	0.0554 (18)	0.0379 (14)	−0.0027 (13)	0.0028 (12)	0.0067 (13)
C8	0.0342 (12)	0.0394 (14)	0.0357 (13)	−0.0002 (11)	0.0024 (11)	−0.0037 (11)
C5	0.0592 (19)	0.082 (3)	0.0438 (17)	−0.0175 (18)	0.0108 (15)	0.0108 (17)
C12	0.089 (3)	0.0383 (18)	0.097 (3)	0.0084 (18)	−0.013 (2)	0.005 (2)
C11	0.0381 (13)	0.0392 (15)	0.0430 (14)	0.0014 (11)	0.0070 (12)	−0.0011 (12)
O3	0.0635 (14)	0.0341 (11)	0.0769 (15)	−0.0017 (10)	−0.0164 (12)	−0.0014 (11)
C10	0.060 (2)	0.061 (2)	0.075 (2)	0.0151 (17)	0.0198 (18)	−0.0072 (18)
C4	0.0387 (14)	0.0532 (17)	0.0517 (17)	−0.0047 (13)	0.0135 (13)	−0.0030 (14)
C6	0.0530 (17)	0.081 (2)	0.0361 (15)	−0.0153 (17)	0.0021 (13)	0.0038 (16)
N1	0.0378 (11)	0.0448 (14)	0.0520 (14)	0.0067 (10)	0.0094 (11)	0.0022 (11)
O1	0.0472 (11)	0.0391 (11)	0.0675 (14)	0.0010 (9)	0.0162 (10)	0.0016 (10)
O2	0.0493 (12)	0.0642 (16)	0.0749 (16)	−0.0133 (11)	−0.0174 (12)	0.0165 (13)
C13	0.0463 (16)	0.0454 (17)	0.0604 (18)	0.0012 (14)	0.0176 (15)	0.0068 (15)
C14	0.0518 (19)	0.094 (3)	0.095 (3)	0.012 (2)	0.030 (2)	0.002 (2)
O4	0.0544 (14)	0.0595 (16)	0.142 (3)	−0.0144 (12)	0.0256 (17)	−0.0311 (18)
O5	0.0482 (12)	0.0809 (17)	0.0622 (14)	0.0056 (11)	0.0182 (11)	−0.0075 (12)
Br1	0.0557 (2)	0.0767 (3)	0.0680 (2)	−0.02186 (17)	0.02031 (16)	0.00019 (19)
C3	0.0383 (14)	0.0518 (17)	0.0452 (15)	−0.0021 (12)	0.0007 (12)	−0.0004 (14)
C7	0.0352 (12)	0.0441 (15)	0.0345 (13)	0.0012 (11)	0.0026 (10)	0.0000 (12)
C9	0.0434 (14)	0.0454 (16)	0.0414 (14)	0.0032 (12)	0.0102 (12)	0.0049 (13)
C1	0.0352 (13)	0.0477 (16)	0.0361 (13)	0.0005 (12)	0.0046 (11)	0.0011 (12)

### Geometric parameters (Å, °)

**Table:**

C2—C3	1.385 (4)	C10—H10A	0.96
C2—C1	1.386 (4)	C4—C3	1.379 (4)
C2—H2	0.93	C4—Br1	1.908 (3)
C8—C11	1.503 (4)	C6—C1	1.382 (4)
C8—C9	1.542 (4)	C6—H6	0.93
C8—C7	1.547 (3)	N1—O1	1.468 (3)
C8—H8	0.98	N1—C7	1.478 (4)
C5—C4	1.367 (5)	O1—C9	1.419 (3)
C5—C6	1.390 (5)	C13—O4	1.181 (4)
C5—H5	0.93	C13—O5	1.326 (4)
C12—O3	1.444 (4)	C13—C9	1.512 (4)
C12—H12C	0.96	C14—O5	1.444 (4)
C12—H12B	0.96	C14—H14C	0.96
C12—H12A	0.96	C14—H14B	0.96
C11—O2	1.202 (4)	C14—H14A	0.96
C11—O3	1.312 (4)	C3—H3	0.93
C10—N1	1.462 (4)	C7—C1	1.505 (4)
C10—H10C	0.96	C7—H7	0.98
C10—H10B	0.96	C9—H9	0.98
			
C3—C2—C1	121.2 (3)	C5—C6—H6	119.5
C3—C2—H2	119.4	C10—N1—O1	104.5 (2)
C1—C2—H2	119.4	C10—N1—C7	113.2 (2)
C11—C8—C9	117.3 (2)	O1—N1—C7	100.31 (19)
C11—C8—C7	108.7 (2)	C9—O1—N1	102.4 (2)
C9—C8—C7	101.9 (2)	O4—C13—O5	125.3 (3)
C11—C8—H8	109.5	O4—C13—C9	126.9 (3)
C9—C8—H8	109.5	O5—C13—C9	107.7 (3)
C7—C8—H8	109.5	O5—C14—H14C	109.5
C4—C5—C6	119.0 (3)	O5—C14—H14B	109.5
C4—C5—H5	120.5	H14C—C14—H14B	109.5
C6—C5—H5	120.5	O5—C14—H14A	109.5
O3—C12—H12C	109.5	H14C—C14—H14A	109.5
O3—C12—H12B	109.5	H14B—C14—H14A	109.5
H12C—C12—H12B	109.5	C13—O5—C14	116.2 (3)
O3—C12—H12A	109.5	C4—C3—C2	118.7 (3)
H12C—C12—H12A	109.5	C4—C3—H3	120.6
H12B—C12—H12A	109.5	C2—C3—H3	120.6
O2—C11—O3	124.4 (3)	N1—C7—C1	113.1 (2)
O2—C11—C8	125.7 (3)	N1—C7—C8	100.8 (2)
O3—C11—C8	109.8 (2)	C1—C7—C8	114.6 (2)
C11—O3—C12	117.1 (3)	N1—C7—H7	109.3
N1—C10—H10C	109.5	C1—C7—H7	109.3
N1—C10—H10B	109.5	C8—C7—H7	109.3
H10C—C10—H10B	109.5	O1—C9—C13	109.1 (2)
N1—C10—H10A	109.5	O1—C9—C8	106.0 (2)
H10C—C10—H10A	109.5	C13—C9—C8	116.8 (2)
H10B—C10—H10A	109.5	O1—C9—H9	108.2
C5—C4—C3	121.5 (3)	C13—C9—H9	108.2
C5—C4—Br1	120.0 (2)	C8—C9—H9	108.2
C3—C4—Br1	118.5 (2)	C6—C1—C2	118.5 (3)
C1—C6—C5	121.1 (3)	C6—C1—C7	119.6 (3)
C1—C6—H6	119.5	C2—C1—C7	121.8 (2)
			
C9—C8—C11—O2	−25.5 (4)	C9—C8—C7—N1	−26.7 (2)
C7—C8—C11—O2	89.3 (3)	C11—C8—C7—C1	87.0 (3)
C9—C8—C11—O3	159.4 (2)	C9—C8—C7—C1	−148.5 (2)
C7—C8—C11—O3	−85.8 (3)	N1—O1—C9—C13	161.6 (2)
O2—C11—O3—C12	−2.7 (5)	N1—O1—C9—C8	35.1 (3)
C8—C11—O3—C12	172.4 (3)	O4—C13—C9—O1	−3.5 (5)
C6—C5—C4—C3	−0.8 (5)	O5—C13—C9—O1	174.0 (2)
C6—C5—C4—Br1	−179.4 (3)	O4—C13—C9—C8	116.5 (4)
C4—C5—C6—C1	0.8 (6)	O5—C13—C9—C8	−65.9 (3)
C10—N1—O1—C9	−170.5 (2)	C11—C8—C9—O1	113.7 (3)
C7—N1—O1—C9	−53.0 (2)	C7—C8—C9—O1	−4.9 (3)
O4—C13—O5—C14	−7.2 (5)	C11—C8—C9—C13	−8.0 (4)
C9—C13—O5—C14	175.2 (3)	C7—C8—C9—C13	−126.6 (3)
C5—C4—C3—C2	0.0 (5)	C5—C6—C1—C2	−0.2 (5)
Br1—C4—C3—C2	178.6 (2)	C5—C6—C1—C7	176.0 (3)
C1—C2—C3—C4	0.7 (5)	C3—C2—C1—C6	−0.6 (5)
C10—N1—C7—C1	−77.9 (3)	C3—C2—C1—C7	−176.7 (3)
O1—N1—C7—C1	171.2 (2)	N1—C7—C1—C6	133.1 (3)
C10—N1—C7—C8	159.2 (2)	C8—C7—C1—C6	−112.1 (3)
O1—N1—C7—C8	48.3 (2)	N1—C7—C1—C2	−50.9 (4)
C11—C8—C7—N1	−151.2 (2)	C8—C7—C1—C2	63.9 (4)

### Hydrogen-bond geometry (Å, °)

**Table:**

*D*—H···*A*	*D*—H	H···*A*	*D*···*A*	*D*—H···*A*
C3—H3···N1^i^	0.93	2.56	3.492 (4)	179
C12—H12C···O1^ii^	0.96	2.52	3.434 (5)	158

Symmetry codes: (i) −*x*+1, *y*+1/2, −*z*+1/2; (ii) *x*, *y*+1, *z*.
